# Automated air-flow cytometry enables real-time monitoring of *Plasmopara viticola* sporangia in vineyards

**DOI:** 10.1128/aem.02152-25

**Published:** 2026-03-30

**Authors:** Amanda Malvessi Cattani, Yanick Zeder, Elias Graf, Andreas Schwendimann, Roberta Coronelli, Tara Smit-Sadki, Jean-Philippe Burdet, Esteban Alfonso, Augustine Jaccard, Erny Niederberger, Markus Rienth

**Affiliations:** 1 University of Sciences and Art Western Switzerland, Changins College for Viticulture and Enology, Nyon, Switzerland; 2 Swisens AG, Emmen, Switzerland; 3 Università degli studi di Bari Aldo Moro, Bari, Italy; The University of Arizona, Tucson, Arizona, USA

**Keywords:** *Plasmopara viticola*, grapevine, SwisensPoleno, downy mildew, precision viticulture, Hirst-type spore trap

## Abstract

Fungal diseases such as downy mildew (*Plasmopara viticola*) can severely affect grapevine yield and quality, making precise fungicide applications critical. Decision Support Systems (DSS) in precision viticulture guide treatment timing, but most rely primarily on weather data and overlook biological indicators, such as real-time spore dynamics. To address this, we evaluated an automatic air-flow cytometer (SwisensPoleno Jupiter) for real-time detection and quantification of *P. viticola* sporangia. The instrument integrates holographic imaging, fluorescence spectroscopy, and AI-based classification. Laboratory data sets (>5,000 events) from naturally and artificially infected leaves enabled the development of classifiers based on Random Forest models. Field validation in Swiss vineyards combined Hirst-type spore traps (microscopy and qPCR DNA quantification) with SwisensPoleno measurements. Correlations between manual and automatic sporangium quantification emerged during a 2023 pilot (60 days) and strengthened over the 2024 season (140 days). Correlations were weak early in the season, during leaf development and the onset of flowering, but improved as vine phenology progressed and disease pressure increased, reaching up to *r* = 0.94 with microscopy counting and *r* = 0.83 with DNA quantification. Additionally, airborne sporangium concentrations were influenced by daily air temperature and the SwisensPoleno instrument inlet height. Visual assessments in 2024 confirmed severe downy mildew, affecting nearly all unprotected leaves, and *P. viticola* DNA concentrations were strongly correlated with symptom severity (*r* = 0.82). With further refinement, these first models for real-time and automatic identification of *P. viticola* sporangia have the potential to provide reliable spore-monitoring data for DSS integration, supporting precision viticulture worldwide.

**IMPORTANCE:**

The automatic, real-time classification of *Plasmopara viticola* spores presented in this study offers a significant advancement in disease risk forecasting. By integrating airborne spore detection, this approach reduces the likelihood of false-positive recommendations, applying fungicides when weather conditions are favorable, but disease risk is low and false negatives, when spores are present, but treatments are not advised. Consequently, this strategy can decrease treatment frequency and overall fungicide use, promoting more sustainable and precise disease management in vineyards.

## INTRODUCTION

Precision viticulture has emerged as a key approach for vineyard management in response to the environmental challenges that winegrowers have faced over the last decade. To provide a clearer understanding of the concept, the International Organization of Vine and Wine (OIV) published in 2019 a resolution defining precision viticulture as a “cyclic management approach to field operations based on information and technology tools that use multiple sources of vineyard-related data to support site-specific decision-making with the aim of optimizing production processes” (1). Different technological tools like sensors, data analytics, and automation are increasingly being adopted by wine growers worldwide. These tools aim to optimize the use of resources such as water, fertilizers, pesticides, energy, and time, thereby reducing costs and minimizing environmental impacts (2).

Disease management strategies have become a major focus of emerging technologies. This is particularly relevant for fungal diseases, where predictive models play a crucial role in minimizing crop losses. Such models rely on mathematical and mechanistic analyses driven primarily by robust weather data and soil characteristics but limited biological information (2). Among the diseases affecting grapevine production, downy mildew, caused by the obligate biotrophic oomycete *Plasmopara viticola*, poses a serious threat in viticulture areas with warm and humid climates (3). All green grapevine tissues, including leaves, inflorescences, fruit clusters, and young bunches, are susceptible to infection by *P. viticola*. Infestation leads to impaired plants’ gas exchange, reduced photosynthetic performance, lower nutrient content and sugar accumulation in berries, diminished bud overwintering capacity, and yield losses (4, 5).

The life cycle of *P. viticola* consists of an asexual multiplication phase during the grapevine vegetative period and a sexual phase that ensures pathogen survival over winter (4). During the growing season, lemon-shaped sporangia release four to eight flagellated zoospores that swim across the water film on the lower leaf surface (6). Nutrients are absorbed from host cells via intracellular haustoria (7). This period of intercellular growth is latent and asymptomatic. After 7–10 days, primary infection symptoms appear as yellowish “oil spots” on the adaxial leaf surface, which later turn necrotic. Masses of hyaline sporangia then emerge from sporangiophores on the abaxial surface, spreading via wind or raindrops. These sporangia initiate secondary infections whenever conditions are favorable, and protection measures are lacking (4).

Neglecting fungicide applications in regions or periods with high disease pressure can lead to total yield loss. In organic viticulture, copper sulfate-based formulations remain the most widely used chemical control method (8). Acupric and systemic fungicides are also applied in conventional viticulture (9). However, due to environmental and health concerns, copper use in organic farming is increasingly restricted across European countries (8). Consequently, research has intensified into alternative approaches, such as biocontrol agents and natural compounds (10–12).

Because *P. viticola* infections depend on weather conditions (temperature, humidity, rainfall) and spore availability, disease pressure can be predicted by models that provide early warnings of potential outbreaks. These models support vineyard management by advising fungicide application strategies based on infection risk levels. In Switzerland, winegrowers benefit from the Agrometeo platform, which provides decision-support tools for improved plant protection. The system integrates data from nearly 200 autonomous weather stations, delivering microclimatic information to drive pest and disease risk models, including those for downy mildew (13). The model incorporates temperature, precipitation, humidity data, modeling-estimated sporangium density, and leaf growth. However, real biological measurements of spore concentrations are not yet implemented.

One of the most widely used strategies for measuring airborne particles relies on a continuous volumetric air sampler that collects samples such as fungal spores and pollen. The sampler type originally described by Hirst in 1952 (14) is still widely employed today, typically with the aid of a pump that draws approximately 10 L of air per minute through a 14 × 2 mm orifice. Behind this entry slot, a rotating drum fitted with a silicon-coated plastic strip captures airborne particles, which adhere to the strip’s surface (15). The drum is replaced weekly, and the collected particles are manually identified and counted under a light microscope. This traditional method is extremely time-consuming, requires considerable expertise, and introduces at least a 7-day delay, representing a major limitation for fungal disease forecasting and decision-support systems. Moreover, accurate identification of fungal spores is further complicated by their high morphological similarity under light microscopy. Molecular approaches, such as quantitative PCR (qPCR) and next-generation sequencing (NGS), have been introduced to overcome these limitations (16). While these methods offer high accuracy, their effectiveness depends heavily on reliable DNA extraction protocols as well as the use of specific probes and primers.

Recently, solutions for automatic and real-time monitoring of airborne particles have been developed (reviewed in Buters et al. 2024 ) (17). One such system, the SwisensPoleno Jupiter, operates on the principle of flow cytometry, integrating digital holographic imaging, laser-induced fluorescence, and depolarization measurement. Ambient air is continuously drawn in at 40 L. min^−1^, and individual particles pass through a narrow measurement channel where they are characterized. First, the system generates holographic recordings that provide information on particle shape and size. Afterward, fluorescence spectral information and lifetime are measured, for which the particles are sequentially illuminated by three light sources (280 nm LED, 365 nm LED, and 405 nm LD). The automatic classification is provided by algorithms and models based on machine learning techniques to assign the isolated target particle to a taxonomic group (family, genus, or species).

In this study, we monitored the seasonal concentration of *P. viticola* sporangia in Swiss vineyards using three complementary strategies: a Hirst-type spore trap (microscopy identification and DNA quantification via qPCR) and the SwisensPoleno system classification. We present the first models for the automatic and real-time identification of *P. viticola* sporangia using the flow cytometer SwisensPoleno, validated against both microscopic and molecular data. In addition, we investigated the impact of SwisensPoleno positioning in the vineyards, the influence of weather conditions on airborne sporangium concentration, and the relationship between airborne sporangia and disease incidence.

## MATERIALS AND METHODS

### Creation of training data sets for *P. viticola* sporangium automatic detection

**Fig 1:**
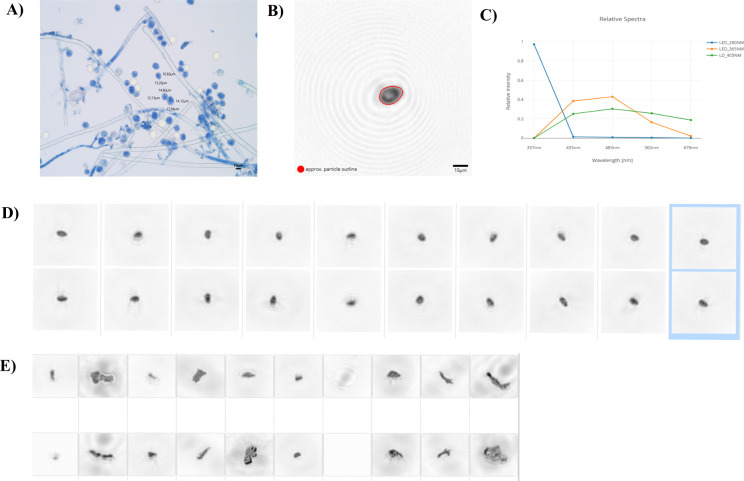
*P. viticola* data set creation using the SwisensPoleno airflow cytometer. (**A**) Light microscopy image of a field population of *P. viticola* sporangia. Mycelium from an infected grapevine leaf was removed and suspended in solution. The cell suspension was mixed with 0.4% trypan blue at a 1:1 ratio and analyzed under a light microscope. Cell length and width were measured. (**B**) Holographic image from the SwisensPoleno instrument of a *P. viticola* sporangium selected based on the feature criteria described in the Materials and Methods. (**C**) Relative fluorescence spectra corresponding to the particle shown in (**B**), obtained using the different fluorescence filter wavelengths employed in this study. (**D**) Examples of holographic images of particles (two for the same particle) associated with *P. viticola* sporangia and integrated into the data set after species fine-grained filtering. (**E**) Examples of holographic images of unwanted particles that were excluded from the final data set.

To develop classification algorithms for the automatic detection of sporangia from *P. viticola* using the SwisensPoleno system, laboratory events data sets were generated. In this context, an event (or measurement event) is the smallest data unit produced by the system. Each particle passing through the SwisensPoleno generates an event, which contains all the measured characteristics of that particle. Specifically, an event includes metadata, a time-resolved light scattering signal, up to two holographic images (captured by the system’s dual cameras), and particle features extracted from the images, fluorescence intensity spectra, and fluorescence lifetime (Fig. 1).

Measurement campaigns were conducted in laboratory conditions during the summers of 2023 and 2024. During these campaigns, the system was supplied with sporangia via controlled nebulization. Fresh sporulating lesions were obtained from grapevine leaves collected in the field as well as from potted vines artificially infected with *P. viticola*. Artificially infected leaves were preferred to reduce unwanted events in the database (for description of methods regarding the artificially infected leaves, see the section *qPCR standards curves and DNA quantification*). Controlled nebulization was performed using pumped air from an adapted medical compressor with a maximum capacity of 10 L.min⁻¹. The air was distributed inside a chamber (approximately 44 cm³) containing the infected leaf and connected to the inlet of the SwisensPoleno instrument, located in the laboratory. The airflow rate was adjusted according to the sporangium density on the infected leaf to maintain a maximum rate of 300 events per minute reaching the SwisensPoleno detector. This approach prevents particles from passing through the detection source too quickly, ensuring a correct capture by the device. The process continued until at least 4,000 events were recorded (approximately 30–40 min), forming what we defined as the raw data set.

Raw data sets were then cleaned based on the specific traits of *P. viticola* sporangia. The following holographic image criteria were used: intensity difference >0.3 (0–1 scale); major axis between 10 and 30 µm; eccentricity > 0.4 (dimensionless); solidity > 0.7 (dimensionless); and equatorial diameter < 20 µm. The intensity feature is related to the grayscale range of the image and was used to remove events without valid image information. The major axis corresponds to particle length and was defined based on morphological observations of multiple *P. viticola* sporangia collected in our fields using microscopy images (Fig. 1A). The same approach was applied to determine the equatorial diameter. Thresholds for eccentricity, defined as the ratio between the focal distance and the major axis length, and solidity, defined as the ratio of pixels in the object region to the pixels of its convex hull, were established during the first campaign. These thresholds were derived from a pool of high-quality holographic images (Fig. 1B and D) in which shape and size parameters (major axis and equatorial diameter) closely matched those observed in microscopy images. After this automated filtering, a second manual curation step was carried out to discard remaining unwanted events (Fig. 1E). Based on these data sets, classification algorithms were developed and subsequently applied to the automated system’s measurement data.

In addition to *P. viticola* data sets, additional species were used for the training. The following list of species was part of the training data: *Alternaria solani*, *Alternaria alternata*, *Fusarium* spp., and *Erysiphe necator*. Moreover, a reference data set was used that is known to have no spores present. This was achieved by extracting measurement events from the system during periods where spores are not present in the air.

### Skeleton-based feature extraction for spore classification

From microscopic observations, it was found that spores exhibit a much greater variety of shapes than pollen. The features extracted by SwisensPoleno so far from holographic images are therefore not sufficient to successfully distinguish spores. This is particularly evident in the case of the frequently elongated forms and long particle chains. To correctly capture these structures and incorporate them into a spore classifier, a skeleton is calculated for each particle image. Skeletonization is an image processing technique in which binary objects are reduced to a characteristic “skeleton line” (18). The particle is simplified as much as possible until only a one-pixel-wide line remains, reflecting the basic structure and topology of the particle.

The resulting skeleton makes it possible to precisely determine features such as the length, width, and branching of the particle. The following skeleton-based properties were used in the classifier: (i) skeleton length, (ii) number of branches, (iii) average distance from the particle outline to the nearest skeleton point, (iv) distance from the particle outline to the nearest skeleton point at multiple evenly distributed points along the skeleton, and (v) minimum and maximum distance from the particle outline to the nearest skeleton point.

These features are then used for the classification and analysis of particles. Skeletonization thus helps to reduce complex shapes to essential structures and enables elongated spores and spore chains to be represented in the classifier, enabling the detection and counting of the number of connected particles (Fig. S1). As the SwisensPoleno records two orthogonal images of each particle, two sets of these skeleton features are extracted. However, to simplify filtering, an attempt was made to merge the two skeletons to extract 3D information about the particle. In particular, an estimated particle length was computed based on the two individual skeletons. This value is then directly comparable to literature references, while the individual skeletons cannot.

### Development of species filters

In addition to the above-mentioned filtering done after the measurement of the particles, an additional filter was implemented. This filter has access to the more advanced skeleton-based values described above, making it possible to have finer-grained control over what should be filtered. The filter has two main functions: (i) making the training data consistent and in line with expected values from literature and (ii) sanity-check data after classification to ensure the predicted class is in line with what the model was trained on.

The first function is important to align data sets from different sources, as it is a way to quickly apply filtering across many data sets and observe the effects. The second function reduces false positives when classifying real-world data. It captures many outliers that are out of distribution of any sample seen during training, therefore making the predictions more stable. The filter specifications for *P. viticola* used in this study are as follows: skeleton branches = 0; particle length along skeleton >15 µm and < 40 µm (based on specific morphological traits of *P. viticola* sporangia as described in the previous section). For training, additional fluorescence-based filters were applied to ensure a clean training data set. Outlier particles were removed when their measured fluorescence spectral ratios differed significantly from those of other particles within the same class. These filters were not applied during time-series inference.

### Training of a Random-Forest-based classifier and post-processing filters

The classifier is based on a Random Forest model (19) and was trained on labeled single-particle data acquired with the SwisensPoleno Jupiter. We used the scikit-learn implementation of the Random Forest classifier with default parameters, except that class weights were set to balanced. Prior to model training, features were standardized using z-score scaling. To further address class imbalance, random oversampling was applied to equalize the number of samples across classes. Twenty percent of the data set was reserved for validation. Model performance on the validation set was evaluated using standard metrics, including the confusion matrix, accuracy, F1 score, and recall. In parallel, high-quality, expert-validated data sets for additional fungi (*Alternaria solani*, *Alternaria alternata*, *Fusarium* spp., and *Erysiphe necator*) associated with important crop diseases were generated by other researcher groups. They used a strategy similar to that employed in this work, with all data generated by the SwisensPoleno instrument. These data sets were then used to validate the *P. viticola* models, ensuring both high data quality and comparability across data sets.

For training, numerous features were extracted, including morphological properties such as particle length, various width measurements along the skeleton, number of branches, as well as fluorescence-based spectral values. The features were normalized with a StandardScaler (scikit-learn) (20) to align different value ranges. The target variable (label) was the respective particle class, which was either manually annotated or assigned by experts. The model was implemented using scikit-learn and trained as an ensemble of many decision trees (Random Forest) to achieve robust classification and avoid overfitting. To reduce false-positive detections, post-processing filters were implemented. These filters define the expected value ranges for a given class or species. The value ranges were derived from the training data. Thus, once a particle has been assigned to a class by the Random Forest algorithm, the corresponding filter for that class is applied. The particle is only counted if it also passes through this filter.

### Field experiments

To evaluate the models developed for the automatic detection of *P. viticola* sporangia, we conducted a pilot test during the late 2023 growing season (August–September) in the vineyards of the Changins College for Viticulture and Enology, Nyon, Switzerland (46°23′58.1″N, 6°13′47.3″E). In this trial, we tested two SwisensPoleno Jupiter aerosol inlet heights: 1.5 m (Pol-1.5) and 2.5 m (Pol-2.5). These heights were selected to correspond to the vine canopy height and to position the inlet above the canopy, respectively. Four instruments (two Pol-1.5 and two Pol-2.5) were deployed across two stations. Station 1 (St1), located at the vineyard edge (first row), hosted Pol61-1.5 and Pol63-2.5, while Station 2 (St2), positioned more centrally, contained Pol62-1.5 and Pol64-2.5 (Fig. S2). Additionally, a manual Hirst-type spore trap (HT) was installed at St2 to serve as a reference (Fig. S2). The 2023 deployment took place in a mixed-variety vineyard collection (*Vitis vinifera* cv. Chasselas, Chasselas violet, Garanoir, and Mourvèdre) used for research and teaching purposes. In the 2024 season, two SwisensPoleno instruments (both equipped with 2.5 m aerosol inlets) were deployed in two zones of another experimental vineyard (*V. vinifera* cv. Chasselas) at Changins (Nyon, Switzerland). One instrument (Pol-NT) was installed in a non-treated (NT) zone, alongside a Hirst-type spore trap (Fig. S3). The second instrument (Pol-T) was placed in a treated (T) zone, where vines were managed according to a conventional fungicide spray schedule (Fig. S3). Concentrations of *P. viticola* sporangia provided by SwisensPoleno are expressed as sporangia per cubic meter of air.

### Hirst-type spore trap monitoring and microscopy counting of *P. viticola* sporangia

In parallel with the SwisensPoleno Jupiter monitoring, a volumetric spore trap (Hirst-type; Burkard Manufacturing Co., United Kingdom) was also deployed to collect airborne particles at the same sites previously described (Fig. S2 and S3). The Hirst-type spore trap was installed at a height of 2.2 meters and operated at an approximately airflow rate of 10 L.min^−1^. Airborne particles were collected through a 14 × 2 mm inlet onto a silicone-coated strip mounted on a rotating drum. The drum rotated at a speed of 2 mm.h^−1^ and was replaced weekly at the same time. The silicone-coated strip was divided into 48 mm segments, each representing 24 h. In the 2024 season, the strip was also cut longitudinally into two equal parts. One half of the tape was used for DNA extraction, and the other half for microscopy analyses. For the 2023 pilot test, DNA quantification was not performed. Each segment was analyzed using an optical microscope (Axio Lab.A1, Carl Zeiss, Germany) with a 40× objective lens. According to the technical specification CEN/TS 16868 (21), which outlines the procedure for analyzing the concentration of airborne pollen grains and fungal spores using a volumetric Hirst-type sampler, around 5% of each segment was scanned by reading two continuous horizontal sweeps. *P. viticola* sporangium counts were expressed as daily average concentrations in particles per cubic meter of air (sporangia.m^−3^). To obtain this value, the number of sporangia counted was multiplied by a conversion factor that accounts for the volume of air sampled (14.4 m³.day^−1^), the sampling area (672 mm²), and the size of the microscope’s field of view at 40× magnification (40×/20).

### DNA extraction and qPCR analysis

After sampling, one half of the silicone-coated strip corresponding to each analyzed day was stored at –20°C until genomic DNA (gDNA) extraction. gDNA from each of the 140 monitored days was individually extracted using the DNeasy PowerSoil Pro Kit (Qiagen, Germany), following the manufacturer’s instructions with minor modifications. Specifically, prior to homogenization, samples were incubated at 65°C for 10 min. Homogenization was then carried out three times (60 s at 6.0 m.s^−1^ with 30 s pauses between cycles) using a FastPrep instrument (MP Biomedicals, USA). Purified DNA was eluted in a final volume of 50 µL and quantified using the Qubit dsDNA HS Assay Kit and Qubit Fluorometer (Invitrogen, USA). To ensure consistency across extractions, two calibrators were included in each batch. These calibrators consisted of silicone-coated strips (same dimensions as the samples) onto which four droplets (20 µL each) of a solution containing 10⁶ *P. viticola* sporangia.mL^−1^ were applied and air-dried at room temperature. Calibrator strips were stored at –20°C until extraction.

Quantitative PCR (qPCR) was performed using a TaqMan assay targeting the ITS1–5.8S rDNA region of *P. viticola,* with two specific primers and a hydrolysis probe (Giop) labeled with FAM (6-carboxyfluorescein) as described previously (22, 23) (Table S1). Reactions were carried out in a 10 µL volume containing: 1× TaqMan Universal PCR Master Mix (Applied Biosystems, USA), 250 nM of Giop_P-FAM probe, 900 nM of each primer (Giop Fw/Rv), and 2 µL of DNA template. Amplification was performed on a Magnetic Induction Cycler (Mic) qPCR system (Bio Molecular Systems, Australia) under the following conditions: 50°C for 2 min, 95°C for 10 min, followed by 40 cycles of 95°C for 15 s and 60°C for 1 min. The samples were analyzed in four technical replicates. Each qPCR plate included a negative control (DNA replaced with commercial nuclease-free water) and a positive control (*P. viticola* DNA at a concentration of 0.007 ng.µL^−1^).

The specificity of the assay for *P. viticola* was validated against common airborne fungal species (24), using strains from the Mycology Research Group of Agroscope–Changins (Nyon, Switzerland) (Table S2). gDNA from these fungi was extracted from 100 mg of fresh mycelium (10-day-old PDA cultures) using the FastDNA SPIN Kit for Plant and Animal Tissues (MP Biomedicals, USA), according to the manufacturer’s protocol. DNA from common airborne fungal species was analyzed by qPCR using the TaqMan Giop assay under the same reaction conditions previously described.

### qPCR standard curves and DNA quantification

To avoid cross-contamination with other grapevine pathogens present in the field, standard curves were established using DNA extracted from sporangia of *P. viticola* propagated under controlled conditions. Sporangia were obtained from artificially infected *V. vinifera* cv. Cabernet Sauvignon plants at the 10–15 leaf stage, following the protocols of inoculation described previously (11, 25). Briefly, all leaves of three plants were inoculated with *P. viticola* by spraying the abaxial surface with a suspension containing 10^6^ sporangia.mL^−1^. Inoculated plants were incubated overnight in darkness at 85% relative humidity (RH) and 20°C and subsequently maintained in climate chambers at 25°C and 80% RH with a 14-h photoperiod. Seven days after inoculation, we observed the first oil spots. Plants were then moistened and incubated for 48 h in darkness to promote sporulation. Freshly sporulating lesions were collected, and sporangium concentrations were determined with a Thomas counting chamber under a light microscope. Genomic DNA was extracted from a suspension of 10⁷ sporangia.mL⁻¹ using DNeasy PowerSoil Pro Kit (Qiagen, Germany) as previously described.

For standard curve preparation, *P. viticola* DNA (0.07 ng.µL^−1^) was serially diluted (1:2, 1:4, 1:10, 1:10^2^, 1:10^3^, 1:10^4^, 1:10^5^, and 1:10^6^). Each dilution was analyzed in four replicates by qPCR using the TaqMan Giop assay under the same reaction conditions previously described. Standard regression curves were generated by plotting the known DNA concentrations against the corresponding CT values measured with the Mic qPCR system (Bio Molecular Systems, Australia). The resulting regression equation was then used to convert experimental CT values into DNA quantities. Sporangium concentrations were expressed as the daily amount of DNA (picograms) per cubic meter of air (sporangia.m⁻³). This value was obtained by multiplying the DNA quantity, as determined from the regression equation, by a conversion factor that accounted for both the sampled air volume (14.4 m³.day⁻¹) and the portion of the sampling area analyzed (half-strip from the hirst-type spore trap).

### Disease assessment and weather data

Downy mildew assessments were performed by the same expert each time and involved the fast and random selection (to avoid rater bias) of a total of 100 grapevine leaves from the middle of the vineyard canopy around the SwisensPoleno at each station. For each leaf, the presence of disease symptoms (oil spots with visible sporulation) was recorded to calculate the percentage of infected leaves. In addition, disease severity was evaluated by estimating the proportion of leaf surface area affected by downy mildew (0%–100%). In the 2024 season, disease assessments were carried out 17 times during the growing season. The timing of these evaluations was guided by infection risk forecasts provided by the Agrometeo Downy Mildew Risk Model for Grapevines (13). In 2023, the disease assessment was performed in August.

Weather data for both 2023 and 2024 were obtained from the Agrometeo platform and from MeteoSwiss (Federal Office of Meteorology and Climatology), using publicly available data sets from the MeteoSwiss Open Data portal. The weather station is located at 46°23′55.4″N, 6°13′51.8″E (Nyon, Switzerland) within the vineyard, approximately 150 m from the instruments (SwisensPoleno and Hirst-type sampler). For this study, the following daily parameters were evaluated: mean temperature (°C), mean wind speed (km.h^−1^), relative humidity (RH, %), total precipitation (mm), and leaf wetness duration (h), the latter estimated from the dew point (13).

Phenological stages of grapevines were determined according to the BBCH scale (an acronym formed from the German names of the coordinating institutions: the Federal Biological Institute Biologische Bundesanstalt, the Federal Variety Office Bundessortenamt, and the chemical industry), which is widely applied to all cultivated plants (26, 27).

### Statistical analysis

Normality of data distribution was assessed using the D’Agostino–Pearson omnibus K² test (*P* < 0.05) in GraphPad Prism software (version 10.6.0). Correlations between weather data and data sets from DNA quantification, Hirst-type spore traps, and SwisensPoleno were assessed using the non-parametric Spearman test (*P* < 0.05), as one or more data sets did not meet the assumptions of normality. When comparisons were based exclusively on SwisensPoleno automatic classifications across different inlet heights and stations, paired t-tests (*P* < 0.05) were applied, as these data sets satisfied the normality assumption. To improve visualization and interpretability, given that the data sets spanned vastly different scales, and to reduce the impact of outliers from daily measurements ranging from 0 to over a hundred spores, data from DNA quantification, Hirst-type spore traps (microscopy counts) and SwisensPoleno were log-transformed. When zero sporangia per day were detected by any instrument, a pseudo-count (*y* = *y* + 1) was applied to all values prior to log10 transformation. All statistical analyses were conducted in GraphPad Prism (version 10.6.0).

## RESULTS

### Training data sets and model development for *P. viticola* sporangium automatic detection

To develop the model for SwisensPoleno automatic detection of *P. viticola* sporangia, we generated 11 data sets from 11 different grapevine leaves: 8 collected in the field and three artificially infected with *P. viticola*. The raw data sets contained over 43,000 events, which, after cleaning, were reduced to about 17,000 high-quality events for algorithm training. This number was subsequently further reduced through the species fine-grained filter to 5,000 measurements.

**Fig 2:**
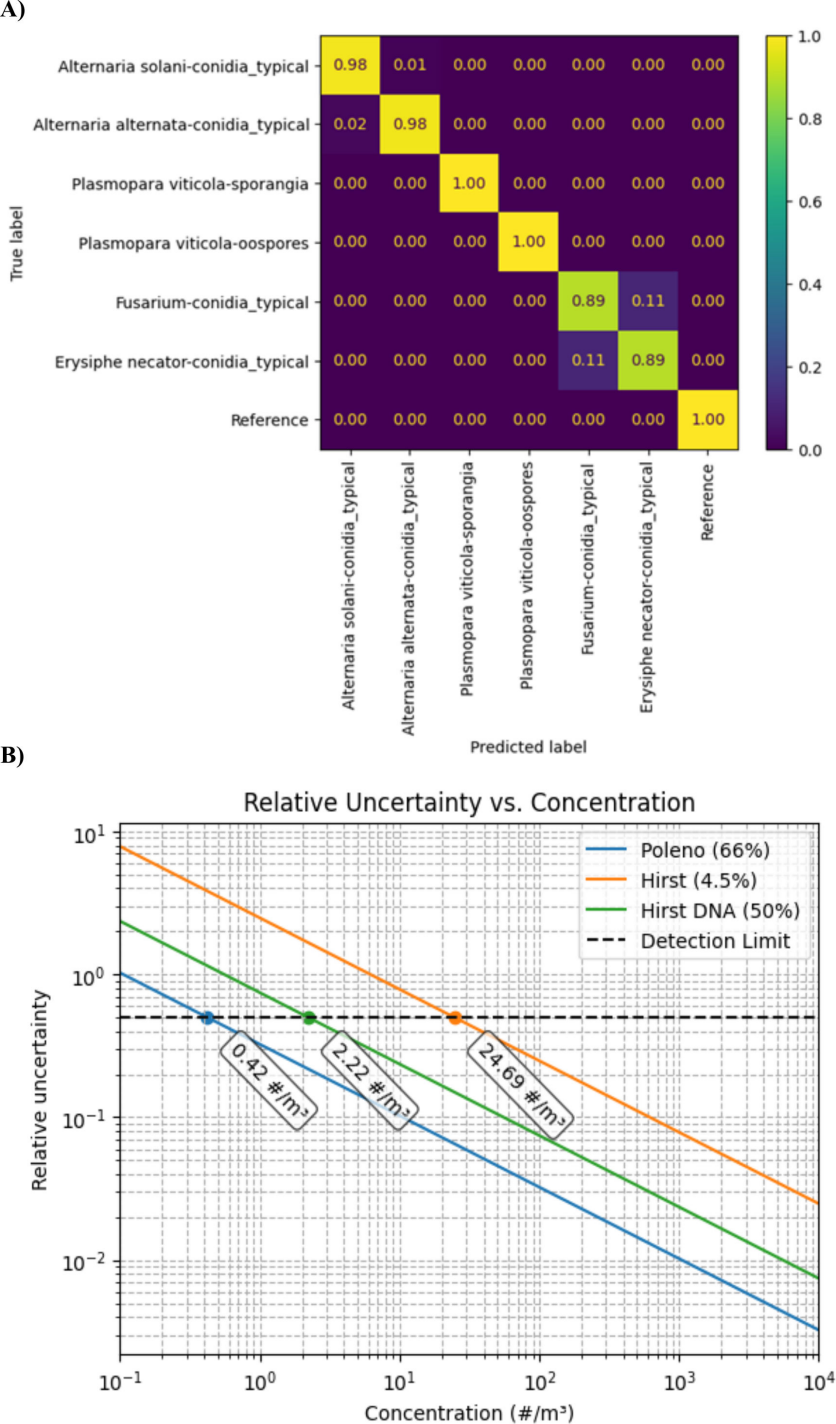
Performance of the *P. viticola* model. (**A**) Confusion matrix for the test set, showing near-perfect classification for most species. The model effectively separated spores from random reference data, demonstrating robust discrimination from background noise. (**B**) Calculation of relative uncertainty (*k* = 2) at a given concentration. Only the statistical error due to sampling is considered, representing a best-case scenario. The detection limit is assumed to be at 50% relative uncertainty as defined in Tummon et al. (28).

The overall model performance (global weighted average across seven classes using 11,200 samples) achieved an accuracy of 0.75, with a precision, recall, and F1-score of 0.73. Furthermore, the classification performance across data sets from other fungal species, summarized in a confusion matrix, demonstrated no misclassification among the other tested species (Fig. 2A). During training, an attempt was made to distinguish between sporangia and oospores, with no success. Furthermore, neither the DNA data nor the Hirst microscope counts could differentiate between these structures. Further work is required to evaluate the model’s ability to distinguish oospores from sporangia.

To enable comparisons of *P. viticola* quantification across different strategies, methodological limitations, such as differences in airflow rate and sampling area that affect detection rates, were taken into account, and correction factors were calculated. For that, sum-scaling was applied by normalizing the seasonal cumulative counts and comparing them to the Hirst reference. Specifically, the ratio was calculated as Correction factor = Σ (SwisensPoleno)/Σ (Hirst-type sampler). Considering that 4.5% of the slides were counted under the microscope, a correction factor of 24.69 was applied to ensure proper comparison with the SwisensPoleno measurements. In addition, since DNA quantifications were performed on 50% of the sampling area of the Hirst-type spore trap, an additional correction factor of 2.2 was applied to the raw quantifications (Fig. 2B).

### The effect of weather conditions, SwisensPoleno positioning, and aerosol inlet height on the detection of *P. viticola* sporangia

During the 2023 pilot season, the classifier developed for the automatic detection of *P. viticola* sporangia was tested using four SwisensPoleno Jupiter with two aerosol inlet heights (Pol61–1.5 m, Pol62–1.5 m, Pol63–2.5 m, and Pol64–2.5 m), deployed in two different vineyard zones (Fig. S2). A manual Hirst-type spore trap was used as a reference. As this was an initial validation of the model and algorithm, monitoring was limited to August and September 2023, covering the phenological stages from fruit development (BBCH 77) to full ripeness (BBCH 87).

**Fig 3:**
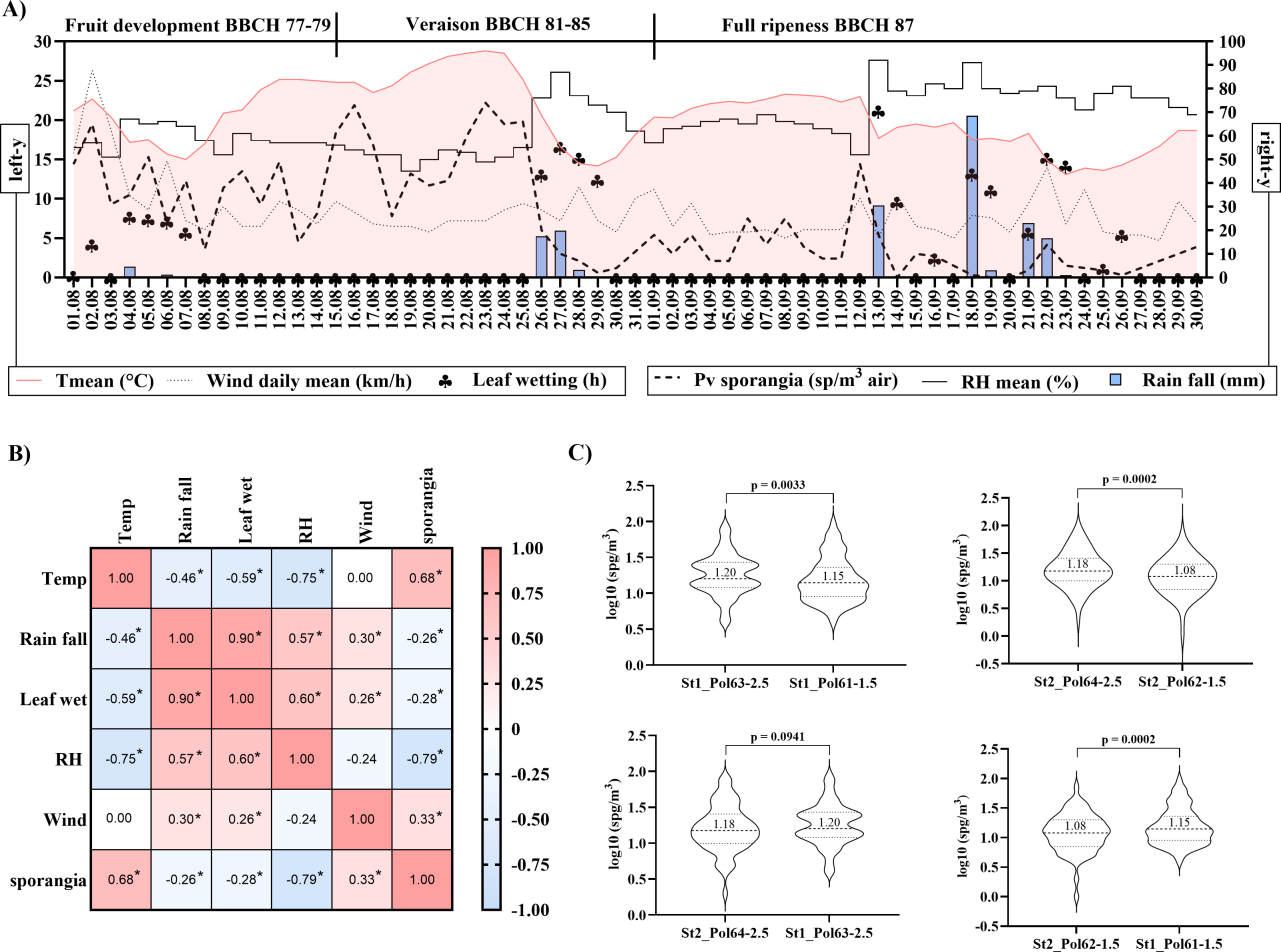
Monitoring of *P. viticola* sporangium concentrations in the air using a manual Hirst-type spore trap and four SwisensPoleno Jupiter. (**A**) Daily weather data were retrieved from the Agrometeo platform and the MeteoSwiss Open Data portal. Concentration of *P. viticola* sporangia measured with the Hirst-type spore trap was manually counted under a light microscope, and concentrations are expressed as sporangia per cubic meter of air. Left y-axis; Tmean, Wind daily mean, Leaf wetting. Right y-axis: Pv sporangia, RH mean, Rainfall. (**B**) Spearman correlations between weather variables and *P. viticola* sporangium concentrations (*n* = 60). Asterisks (*) indicate statistically significant correlations (*P* < 0.05). (**C**) Daily measurements from SwisensPoleno (Pol) instruments at two inlet heights (Pol61–1.5 m, Pol62–1.5 m, Pol63–2.5 m, and Pol64–2.5 m) were compared using paired t-tests (*n* = 60; *P* < 0.05). Medians and calculated *P*-values for each data set comparison are shown. Abbreviations: Tmean, daily mean temperature; RH, relative humidity; Pv, *P. viticola;* sp, sporangia; St, Station 1 or 2.

Weather conditions differed markedly between the 2 months. August 2023 was characterized by lower precipitation and higher temperatures, resulting in reduced relative humidity (RH), compared to September (Fig. 3A). Daily spore counts from the Hirst-type trap revealed more frequent peaks of *P. viticola* sporangia in August than in September (Fig. 3A). This seasonal difference was consistent with the strong positive correlation between airborne sporangium concentration and temperature (*r* = 0.68), and the strong negative correlation with RH (*r* = –0.79; Fig. 3B).

We also investigated the influence of aerosol inlet height on sporangium detection. At 2.5 m inlet height, sporangium detection was consistent across both vineyard zones (St1 and St2), suggesting that sensor location within the vineyard did not significantly affect measurements (*P* = 0.0941; Fig. 3C). In contrast, differences between stations were observed when an inlet height of 1.5 m was used, with a 6% increase in the median sporangium concentration at St1 when considering all analyzed days. When different inlet heights were compared within the same station, daily sporangium concentrations also varied, indicating that aerosol inlet height has a measurable effect on detection. Considering the median across all measurement days, sporangium quantification decreased by 5% and 8% at St1 and St2, respectively, when the SwisensPoleno aerosol inlet was positioned at 1.5 m compared with 2.5 m (Fig. 3C).

**Fig 4:**
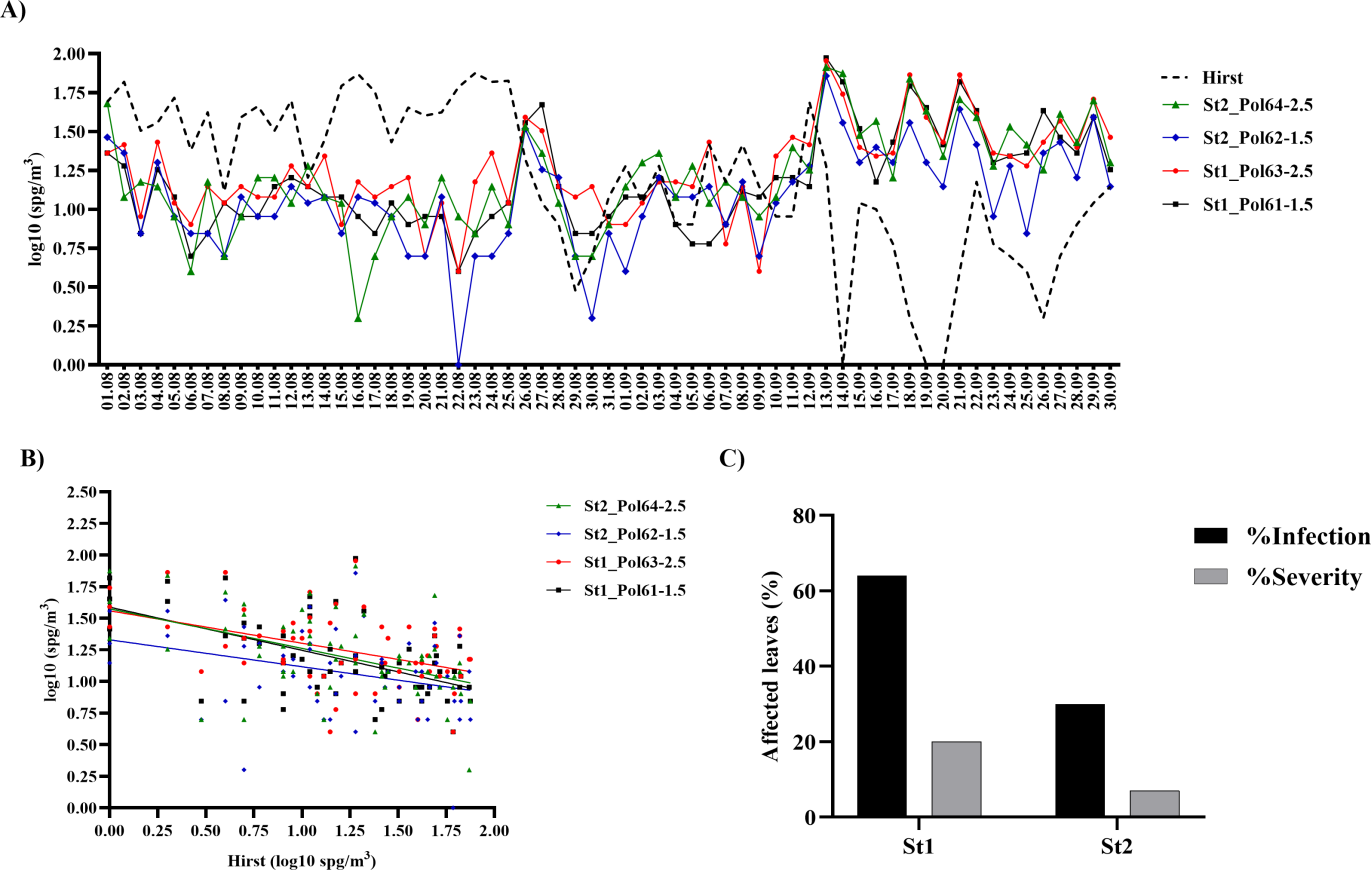
Comparison of SwisensPoleno automatic classifications from different stations with manual Hirst-type spore trap measurements during the 2023 season. (**A**) Daily concentrations of *P. viticola* sporangia (spg) per cubic meter of air (log₁₀-transformed) from all SwisensPoleno instruments (Pol61–1.5 m, Pol62–1.5 m, Pol63–2.5 m, and Pol64–2.5 m) and Hirst-type spore traps at both vineyard stations (St1 and St2) were analyzed over the 60 days of the 2023 pilot test. (**B**) Simple linear regressions were performed to compare data sets. (**C**) A disease assessment was carried out once during the 2023 pilot test on vines surrounding the SwisensPolenos, recording the percentage of leaves with downy mildew symptoms (% infection) and the percentage of leaf surface area affected (% severity).

A comparison between microscope manual counts obtained with the Hirst-type spore trap and automatic classifications produced by the SwisensPoleno revealed a weak correlation during the initial 2023 tests, with coefficients of determination (*R*²) of 0.22, 0.10, 0.19, and 0.30 for St2_Pol64-2.5, St2_Pol62-1.5, St1_Pol63-2.5, and St1_Pol61-1.5, respectively (Fig. 4A and B). Higher concentrations of *P. viticola* sporangia were detected in August 2023 using the Hirst-type sampler compared with the SwisensPoleno, including several concentration peaks that were not captured by the automatic system. In contrast, during the second half of September, peaks in *P. viticola* concentrations were detected by the SwisensPoleno but not by the Hirst-type sampler, which likely explains the overall poor correlation observed between the two methods. Additionally, the Hirst sampler exhibits a relative uncertainty of approximately 80% at concentrations of 10 particles m⁻³. Even at the maximum concentration recorded in 2023 (~60 m⁻³), the relative uncertainty remains around 30%. This high level of uncertainty makes it particularly challenging to validate any correlation for this season, indicating the need for further efforts on classifier improvement.

The internal consistency of the SwisensPoleno unit measurements was confirmed, with significant positive correlations of at least 0.71 between separate units (Fig. S4)

The disease assessment conducted during the 2023 pilot test revealed that vines at St1 had 64% of leaves affected and 20% of the leaf surface area showing downy mildew symptoms. At St2, 30% of leaves were infected, with an average of 7% of their surface area affected (Fig. 4C).

### DNA-based detection of *P. viticola*: standard curve and qPCR specificity

To increase the precision and enable direct comparison of the automatic detection of sporangia by the SwisensPoleno, we also quantified the DNA concentration in the silicone-coated strips collected with the Hirst-type spore trap. The same sample (longitudinally divided) was used for both microscopic counting and DNA quantification of *P. viticola* sporangia. For this purpose, standard curves were generated from DNA extracted from a suspension of 10⁷ sporangia.mL^−1^ (22). Under our conditions, *P. viticola* DNA was detectable across a range from 0.1 ng to 0.01 pg. The resulting standard curve showed an excellent fit to a linear regression, with an *R*² of 0.9949 (Fig. S5). In the specificity test for *P. viticola*, the Giop probe/primer set consistently amplified purified *P. viticola* DNA, while no amplification was detected in purified DNA from common airborne non-target organisms (Table S2).

### *P. viticola* sporangium detection over an entire season: automatic classification, microscopy counts, and molecular quantification

Throughout the 2024 season, we monitored the daily concentration of airborne *P. viticola* sporangia using four complementary approaches. Air samples were collected from Swiss vineyards planted with *V. vinifera* cv. Chasselas from leaf development (BBCH 14) to berry ripening (BBCH 89).

**Fig 5:**
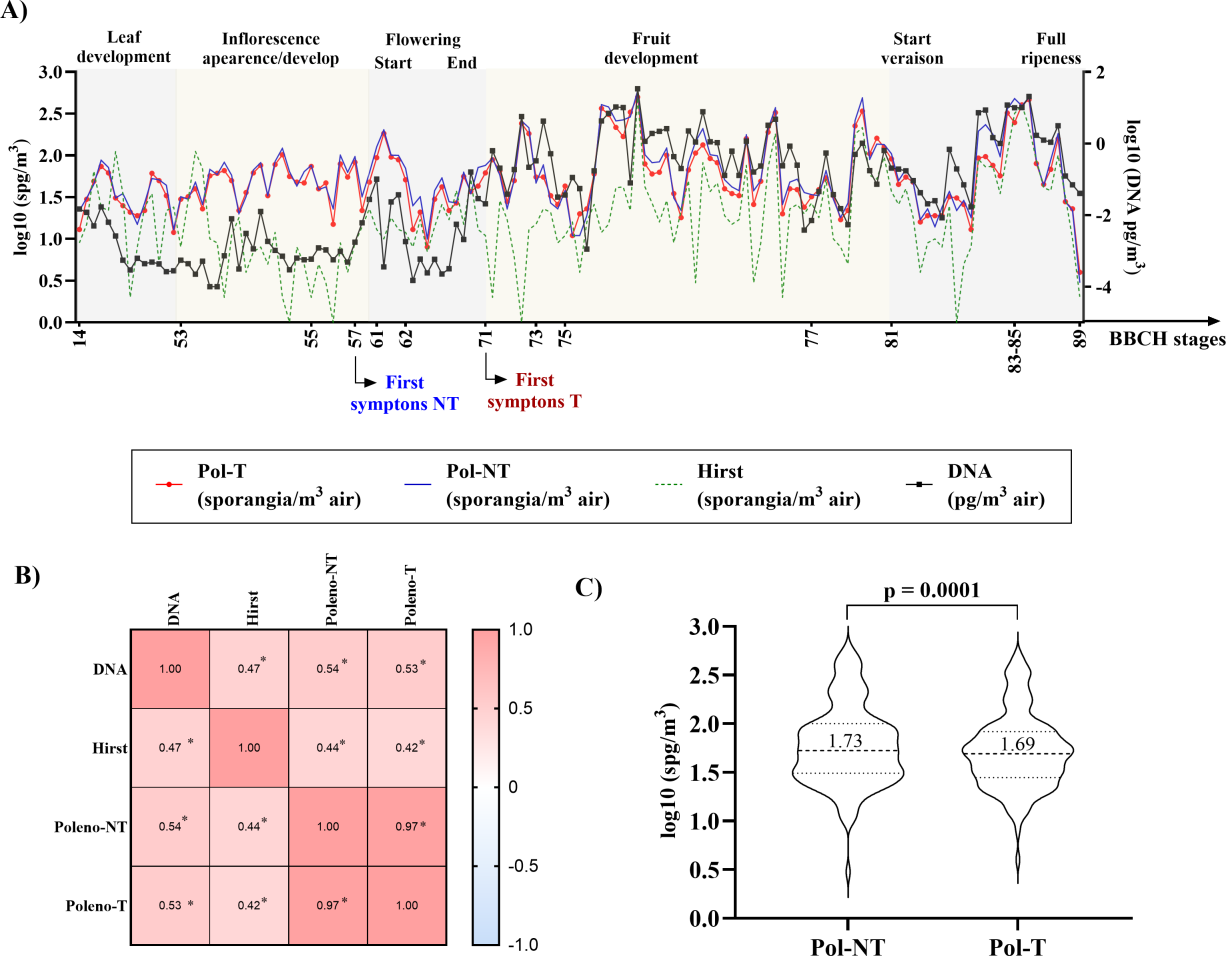
Monitoring airborne *P. viticola* sporangia during the 2024 season using four complementary approaches. (**A**) Daily sporangium concentrations (log₁₀-transformed; *n* = 140). Left y-axis: data sets from Pol-T, Pol-NT, and Hirst represented by sporangia (spg) per cubic meter of air. Right y-axis: data set from daily quantification of *P. viticola* DNA (picograms per cubic meter of air). Phenological stages are annotated according to the BBCH scale. The observation of the first symptoms of downy mildew disease is marked in non-treated (NT) and fungicide-treated (T) plots. (**B**) Spearman correlations (*n* = 140) between automatic classifications from SwisensPoleno (Pol) instruments (Pol-NT, Pol-T), microscopic counts (Hirst), and DNA quantification, covering the period from leaf development to full ripeness. Asterisks (*) indicate statistically significant correlations (*P* < 0.05). (**C**) Daily measurements from SwisensPoleno placed in fungicide-treated (T) and untreated (NT) vineyard zones, compared using paired t-tests (*n* = 140; *P* < 0.05). The medians and the calculated *P*-value for this comparison are also shown.

As expected, an increase in airborne *P. viticola* sporangia coincided with the first visible field symptoms of downy mildew, a period that overlapped with flowering. During this stage, DNA concentrations of *P. viticola* increased markedly (Fig. 5A).

Across the entire monitoring period (140 days), we observed a significant positive correlation between DNA quantification and microscopic counts obtained from the Hirst-type spore trap (*r* = 0.47). A slightly stronger correlation was found with automatic classifications from the SwisensPoleno (Pol-NT: *r* = 0.54; Pol-T: *r* = 0.53; Fig. 5B). In 2024, correlations between the Hirst trap and SwisensPoleno were slightly weaker (Pol-NT: *r* = 0.44; Pol-T: *r* = 0.42) than those observed between SwisensPoleno and DNA (Fig. 5B).

Furthermore, SwisensPoleno sensors positioned in the treated and untreated vineyard zones detected different daily airborne sporangium concentrations, with median concentrations higher in the untreated zones (Fig. 5C). Nevertheless, a strong correlation between the two instruments was observed (*r* = 0.97), and similar correlations were observed when comparing DNA quantification (*r* = 0.53; 0.54) and microscopic counts (Hirst) (*r* = 0.42, 0.44) across the Pol-T and Pol-NT instruments, respectively (Fig. 5B).

**Fig 6:**
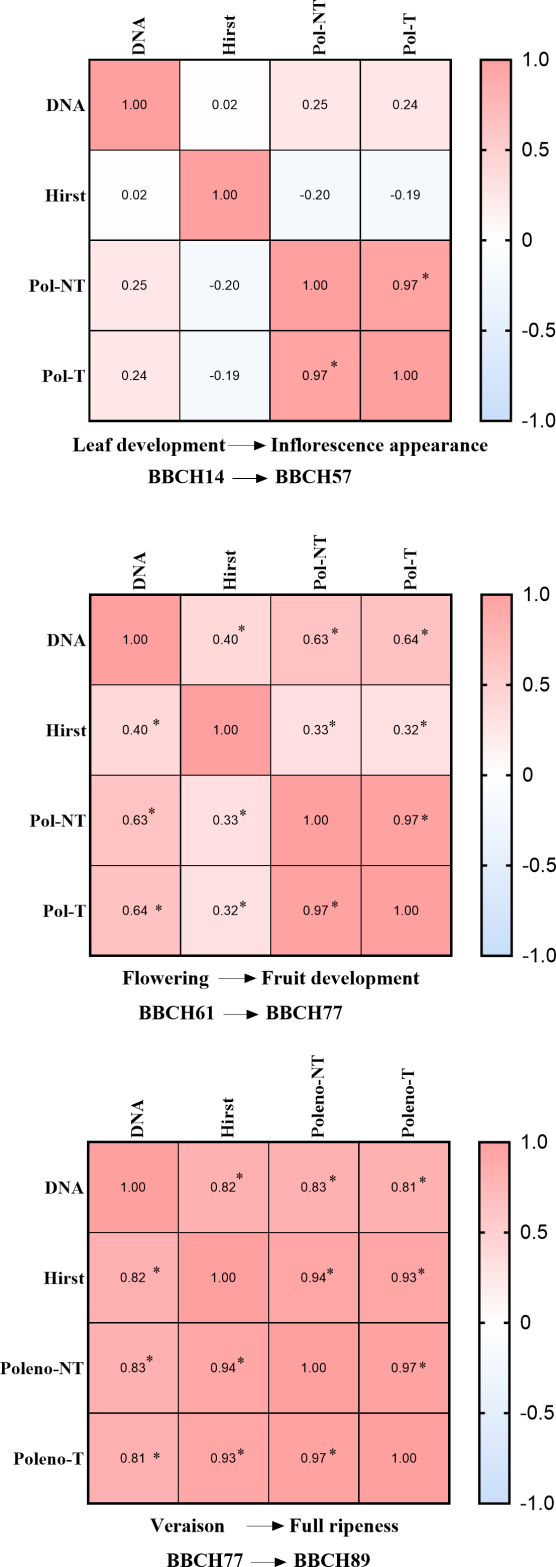
Correlation of measurements from SwisensPoleno, DNA quantification, and manual microscopic counting across vine phenological stages. Spearman correlations were calculated using log₁₀-transformed data from daily quantification of airborne *P. viticola* sporangia obtained by automatic classification with SwisensPoleno (Pol-NT and Pol-T), manual microscopic counts (Hirst), and molecular analyses (DNA). Three data sets were analyzed: 42 days from leaf development to inflorescence emergence, 59 days from flowering to fruit development, and 39 days from veraison to berry ripening. Phenological stages were classified according to the BBCH scale. Asterisks (*) indicate statistically significant correlations (*P* < 0.05).

Considering the phenological stages of the vines, no significant correlations among DNA quantification, microscopic counts (Hirst), and automatic classifications (Pol-NT and Pol-T) were observed during the initial 42-day period from leaf development (BBCH 14) to inflorescence emergence (BBCH 57; Fig. 6), likely due to the low concentration of airborne *P. viticola* sporangia at this stage (Fig. 5). Correlations improved markedly during the subsequent 59 days, spanning flowering (BBCH 61) to fruit development (BBCH 77). During this period, the Spearman correlation of DNA quantification reached *r* = 0.40 for the Hirst-type sampler, *r* = 0.63 for the Pol-NT instrument, and *r* = 0.64 for the Pol-T instrument (Fig. 6).

In the final 39 days, covering veraison (BBCH 77) through to berry ripening, correlations were even stronger. Automatic classifications by Pol-NT and Pol-T showed robust associations with *P. viticola* DNA concentrations (*r* = 0.83 and 0.81) as well as with Hirst manual counts (*r* = 0.94 and 0.93), respectively (Fig. 6). Linear regression analyses followed the same trend, yielding *R*² values of 0.59, 0.66, and 0.61 when DNA quantification was compared with Hirst counts, Pol-NT, and Pol-T, respectively, during the veraison and berry ripening stage (Fig. S6).

### Airborne dynamics of *P. viticola* sporangia in relation to weather conditions during the 2024 season

**Fig 7:**
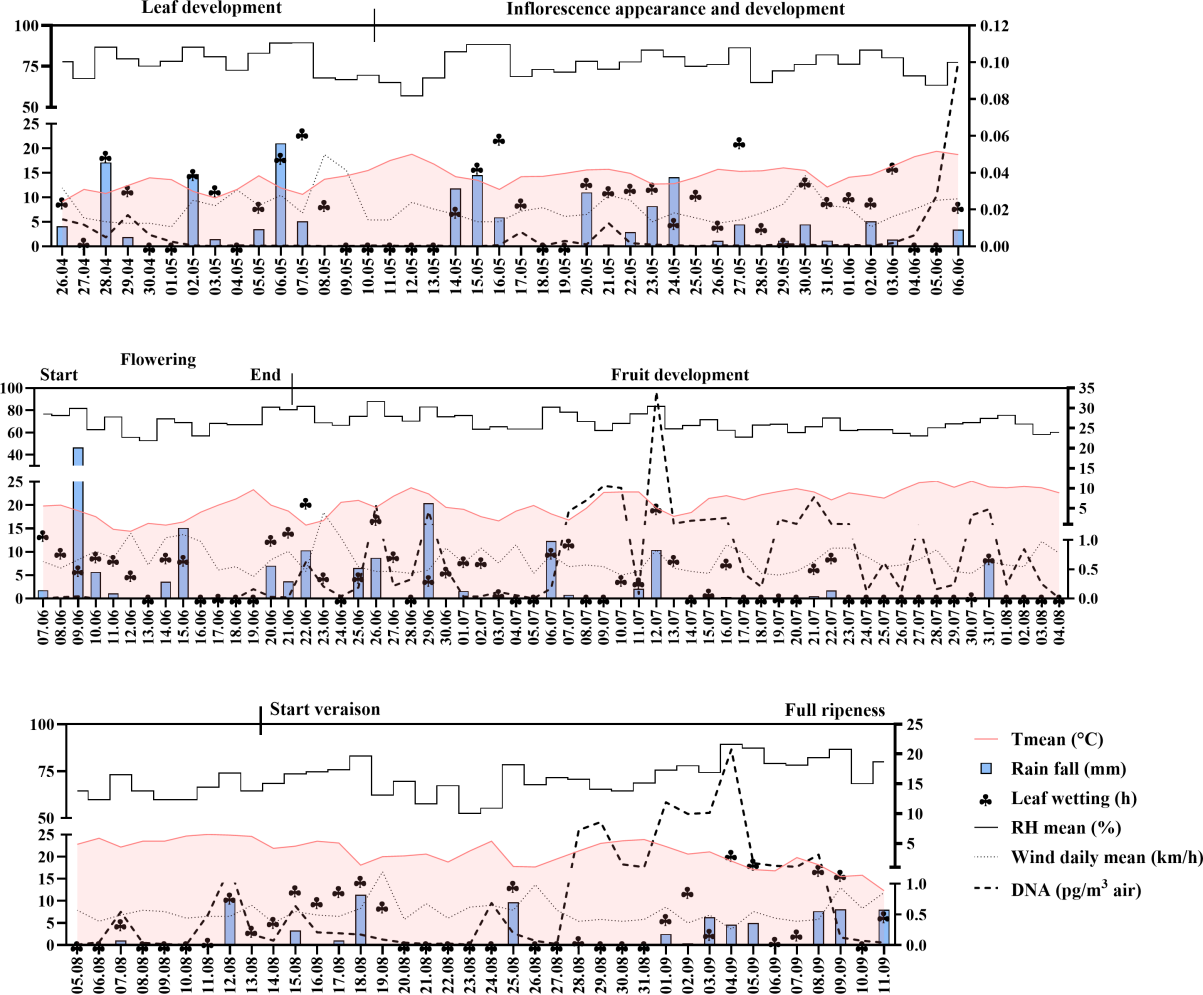
Seasonal dynamics of *P. viticola* sporangia in the air during the 2024 season. Daily weather data were obtained from the Agrometeo platform and the MeteoSwiss Open Data portal. DNA concentrations, evaluated via qPCR, are expressed as picograms of DNA per cubic meter of air. Left y-axis: daily mean temperature (Tmean; °C), wind speed (daily mean; km/h), leaf wetness (hours), relative humidity (RH mean; %), and rainfall (mm). Right y-axis: DNA concentration (pg, sporangia).

During the 140 sampling days from late April to mid-September, peaks in *P. viticola* airborne DNA were typically observed following rainfall events, when foliage remained wet (Fig. 7). In May, only a few oil spots of downy mildew with limited sporulation were detected at the monitored sites, which explains the low concentrations of *P. viticola* in the air. The first disease symptoms appeared in early June on vines surrounding the SwisensPoleno and Hirst-type spore traps. Shortly thereafter, airborne sporangium levels increased markedly.

Concentrations remained low during the early phenological stages, including leaf development and inflorescence onset, but pronounced peaks occurred during fruit development, with the highest concentrations recorded in mid-July. A second major peak of airborne *P. viticola* sporangia was observed in late August to early September (Fig. 7).

Among the weather parameters, air temperature showed the strongest correlation with airborne *P. viticola* DNA concentrations (*r* = 0.64), a pattern consistently observed in both the 2023 and 2024 seasons (Fig. 3B; Fig. S7). In contrast, relative humidity had no clear impact on sporangium levels in 2024 (Fig. S7), and the other parameters showed no significant correlations with the presence of airborne *P. viticola* sporangium DNA.

### Airborne *P. viticola* sporangium and downy mildew incidence in monitored vineyards

**Fig 8:**
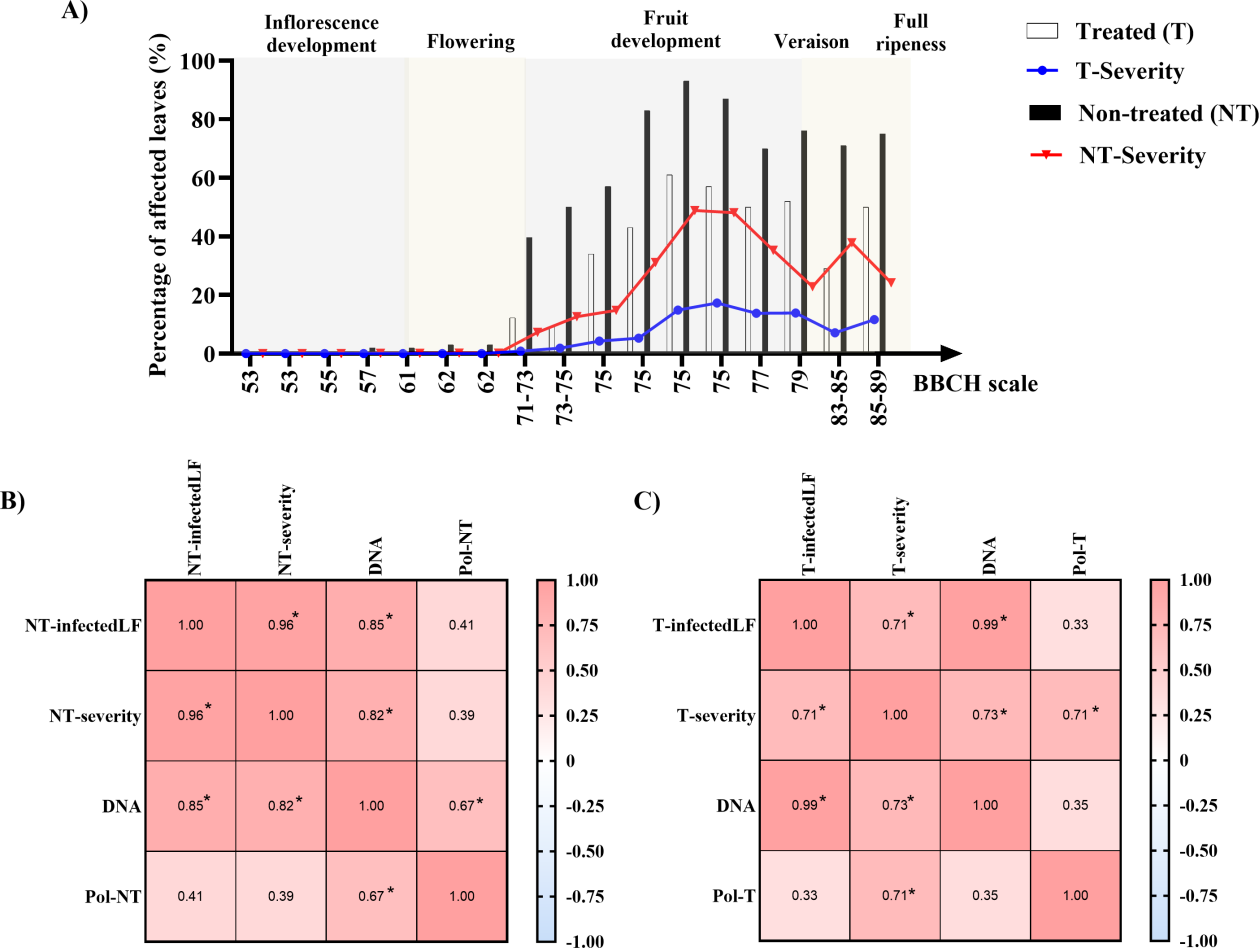
Correlation between airborne *P. viticola* sporangium concentrations and downy mildew disease incidence and severity in treated (T) and non-treated (NT) vineyard plots. (**A**) Field observations of disease incidence (percentage of leaves showing downy mildew symptoms (white bars for treated plots and black bars for non-treated plots) and disease severity (blue and red lines, for T and NT vines, respectively). Spearman’s correlation was calculated (*P* < 0.05) between measurements of airborne *P. viticola* sporangium concentration obtained with the SwisensPoleno system located in non-treated (Pol-NT) (**B**), and treated plot (Pol-T) (**C**) and compared with *P. viticola* DNA quantification and downy mildew disease Incidence (infectedLF) and severity in the respective plot. Asterisks (*) indicate statistically significant correlations.

In the 2024 season, high disease pressure was observed. In non-treated plots (NT), vines were severely affected by downy mildew, with nearly 100% of leaves showing symptoms and high disease intensity (Fig. 8A). In contrast, treated plots (T) exhibited significantly reduced disease severity, although infection was still evident. A strong positive correlation was found between airborne sporangium concentration and disease incidence in treated plots, whereas in non-treated plots, sporangium levels correlated more closely with symptom severity than with incidence alone (Fig. 8B and C).

## DISCUSSION

The development of machine learning (ML) models relies on high-quality data sets, and data filtering is a critical step to ensure that appropriate examples are used for training. The data set creation in this study was guided by a literature review and field observations, through which specific morphological and fluorescence features characteristic of the target organism were identified (29) and then translated into measurable parameters within the SwisensPoleno system (Fig. 1). Among supervised algorithms, the Random Forest model is widely recognized as a powerful technique for species distribution modeling (30, 31). Accordingly, the strong performance of Random Forest models was also confirmed in this work, with the model reaching an accuracy of 0.75 and a specificity of 1.0 among other tested fungi (Fig. 2). High predictive accuracy is particularly important in this context, as false negatives could seriously compromise downy mildew management by leading to missed fungicide applications and, consequently, reduced grapevine protection. False positives, however, may still occur, especially when two classes share highly similar properties, making misclassification unavoidable. In such situations, integrating complementary molecular diagnostic tools would strengthen detection confidence. For example, rapid LAMP (loop-mediated isothermal amplification) assays targeting the ITS region of *P. viticola* have demonstrated high specificity and sensitivity. These assays enable the detection of latent and visible infections in grape leaves. When combined with a rapid DNA extraction protocol, the method becomes suitable for on-site field applications (32). Another frequent source of error is the presence of outliers, particles whose characteristics deviate markedly from those represented in the training data set. Because only a subset of all possible particles can be included during training, such outliers commonly arise in real-world data. Therefore, a robust number of particles is crucial for training the models, ensuring that biological variability is represented as fully as possible. It is also important to note that classifier performance evaluated on laboratory-based training and test data sets is often overly optimistic compared with real-world conditions. Controlled environments cannot fully reproduce the diversity of interfering particles or the natural variability of fungal spores encountered in the field. Consequently, accuracies reported under laboratory conditions do not necessarily reflect operational performance. To address this limitation, we performed independent validation using a Hirst-type volumetric spore trap for microscopic identification, together with DNA-based analyses. These complementary approaches provide a more realistic assessment of the classifier’s ability to detect and quantify fungal spores under field conditions.

When comparing results obtained from different airborne spore measurement strategies, methodological limitations must also be considered (Fig. 2). Microscopy-based identification is a common and widely used technique for spore counting; however, it is prone to misclassification due to the similarity in shape and size among various spores and pollen under light microscopy. Furthermore, the high particle density on silicone-coated strips collected over 24 h makes it impractical to analyze the entire sampled surface. Therefore, statistical studies are necessary to determine the minimum area required to obtain representative results (21). qPCR performance can be limited by false negatives and reduced sensitivity caused by environmental contaminants (PCR inhibitors) such as phenols, humic and fulvic acids, polyphosphates, heavy metals, plant polysaccharides, or excessive non-target DNA (33, 34). Sensitivity is also affected by genetic variability in the target organism, since sequence differences in primer or probe-binding regions can reduce amplification efficiency.

Besides its limitations, qPCR is one of the most sensitive and specific methods for detecting fungal species in air samples. This approach is rapid and eliminates the need for post-PCR electrophoresis (35). In this work, we quantified airborne *P. viticola* sporangia by qPCR using the TaqMan fluorogenic and species-specific hybridization probe system, as described previously (22, 23). To the best of our knowledge, this is the first report to combine this methodological approach with air sampling using a Hirst-type spore trap for *P. viticola* sporangium detection. A recent study in India used qPCR with SYBR Green to detect airborne *P. viticola* sporangia (36). While SYBR Green is more cost-effective and simpler to use (detecting all double-stranded DNA), TaqMan probes offer higher specificity and enable multiplexing, providing superior accuracy, particularly when non-specific amplification products may occur.

Although both microscopic identification and DNA quantification were performed using material collected from the same source (the 48 mm-long Hirst-type strip, divided into two equal parts), they do not correspond to exactly the same sample. This is particularly evident when airborne particle density is low. Under these conditions, particle distribution along the strip becomes more random and non-homogeneous compared to days with high particle density, resulting in weaker correlation scores. Additionally, each sporangium can contain between 4 and 8 zoospores (6), which makes the direct comparison between the number of *P. viticola* sporangia and DNA quantification difficult. This challenge is especially pronounced when very few sporangia are detected.

During the 2023 pilot test, correlations between manual microscopic identification of *P. viticola* and the automatic classifications generated by the SwisensPoleno were difficult to establish (Fig. 4). Similarly, throughout the 2024 grapevine growing season, only a weak correlation was observed among the different methods used to quantify airborne *P. viticola* sporangia, from leaf development to inflorescence emergence (Fig. 5 and 6). Several factors may explain this observation. For instance, comparisons of SwisensPoleno measurements at different aerosol inlet heights revealed daily variations. Since primary infections originate from oospores present in leaf litter near the ground, positioning air samplers at higher elevations may not be optimal during the early season. Likewise, Muthukumar et al. 2025 reported that spore traps installed at 0.5 m detected higher sporangium counts than those placed at 2.5 m (36). Considering that the lowest sampling height in this study was 1.5 m, and that data on the flight dynamics of *P. viticola*, particularly during primary infections, are still limited, it is possible that the particles were not effectively captured by the inlets of either the SwisensPoleno or the Hirst-type spore trap.

The low detection of *P. viticola* sporangia early in the season could also be related to the low air temperatures in Switzerland during this period, which are unfavorable for sporangium dispersion. This hypothesis is supported by our data showing that higher air temperatures correlated with increased sporangium concentrations (Fig. 3 and 7; Fig. S7). Nonetheless, further improvements are needed to optimize early-season detection thresholds. Potential strategies include adjusting the Poleno inlet height, starting lower at the beginning of the season, and gradually increasing it as disease pressure rises. Canopy architecture throughout the season is another important factor that could be used to optimize instrument positioning. Moreover, implementing an early qualitative assay, such as the LAMP-based molecular diagnosis, that can subsequently be complemented by precise sporangium concentration measurements could further support the monitoring of early downy mildew infections. Additionally, enhancing the differentiation between sporangia and oospores could improve the timely identification of disease onset.

Muthukumar et al.(36) made similar observations, noting a sharp decline in spore counts from the three-leaf to inflorescence emergence stage, likely due to reduced sporulation or limited inoculum availability. As oospores mature, sporangia emerge and vine phenological stages progress, more leaves with developed stomata become susceptible to downy mildew infection, leading to a rapid increase in sporangium concentration and improved correlation between measurement strategies (Fig. 6 and 7). This observation is consistent with the evaluation of treated and non-treated plots for disease incidence, where disease spread and intensity were strongly and positively correlated with the concentration of airborne *P. viticola* sporangia (Fig. 8).

Moreover, our results revealed significant variation in daily *P. viticola* sporangium concentrations across different stations, highlighting the importance of strategically placing SwisensPoleno sensors in commercial vineyards. For instance, in 2023, the St1 vineyard plot showed higher median sporangium concentrations per cubic meter compared to St2. Consistently, vines in St1 also exhibited a higher incidence of downy mildew (Fig. 3 and 4). Similarly, in 2024, untreated plots with a higher number of vine disease lesions recorded greater daily sporangium concentrations than fungicide-treated plots (Fig. 5). The SwisensPoleno operates fully automatically, requiring only annual maintenance and can be remotely monitored and controlled. The area effectively monitored by a single sensor depends on several factors, including local bioaerosol transport, type of spore, geographical location, and surrounding landscape. The dispersal range of airborne plant pathogenic spores varies widely. For instance, spores of *Phytophthora infestans* (potato late blight), wheat stem rust (*Puccinia graminis*), wheat stripe rust (*Puccinia striiformis*), and wheat leaf rust can travel tens to thousands of kilometers (37). Similarly, soybean rust (*Phakopsora pachyrhizi*) and other downy mildew pathogens (*Peronospora* spp.) can disperse over large distances. In such cases, a single measuring station can cover an area with a diameter of up to 10 km. For pathogens that spread only locally, it is possible to place the sensor at a representative, high-risk location, serving as an early warning station for the surrounding region.

With further refinement, these automated monitoring tools can provide reliable spore detection data and be integrated into decision support systems (DSS). Integration is particularly relevant given the proven potential of DSSs to optimize crop disease management. For example, Lázaro et al. (38) evaluated 80 independent experiments across 22 studies comparing calendar-based and DSS-guided fungicide application strategies. Their analysis showed that the median number of fungicide applications was 43% lower under DSS-based programs, while achieving equal or higher disease control efficacy (up to 5.5%). The advantage of DSSs was especially pronounced when spray frequencies were low (<4 applications), whereas both strategies performed comparably at higher frequencies. Taken together, these refinements would strengthen the model’s utility for early-warning monitoring and support more informed disease management in vineyards.

### Conclusion

In this study, we present the first machine learning–based models for the automatic, real-time detection of *P. viticola* sporangia using the SwisensPoleno Jupiter system. High-quality data sets were generated and used to train the model, which demonstrated strong performance under laboratory conditions, with an accuracy of 0.75 and specificity of 1.00 when confronted with common airborne fungal particles. Field validation encompassed the monitoring of seasonal dynamics of airborne sporangia throughout the growing season using both Hirst-type spore traps and the SwisensPoleno instrument. Validation via DNA quantification and microscopic identification showed strong correlations, up to 0.9 under high disease pressure, confirming the reliability of the automatic detection system. Notably, the model performed particularly well from the first appearance of downy mildew symptoms, revealing that further refinements or complementary strategies are needed to detect latent infections.

Our results also highlight the influence of sensor placement and aerosol inlet positioning on daily measurements of *P. viticola* sporangia. Sites with higher disease pressure consistently recorded greater sporangium concentrations, whereas sites with lower symptom levels showed fewer airborne spores. These findings emphasize the importance of strategic sensor deployment in vineyards and demonstrate the potential of integrating real-time spore detection into disease management strategies. Overall, this study establishes a robust framework for automated, field-based monitoring of downy mildew, paving the way for more precise and timely interventions in viticulture downy mildew disease management.

## Data Availability

Raw data presented in this study are available upon request from Swisens AG (https://www.swisens.ch/en/contact). The obtained concentrations of *P. viticola* sporangia measured with the Swisens Poleno are available in Zenodo at https://doi.org/10.5281/zenodo.19323693.

## SUPPLEMENTAL MATERIAL

Supplemental material aem.02152-25-s0001.pdf Fig. S1 to S7; Tables S1 and S2.

ASM does not own the copyrights to Supplemental Material that may be linked to, or accessed through, an article. The authors have granted ASM a non-exclusive, world-wide license to publish the Supplemental Material files. Please contact the corresponding author directly for reuse.
