# Supplementary material for: Automated air-flow cytometry enables real-time monitoring of Plasmopara viticola sporangia in vineyards
Source: Appl Environ Microbiol. 2026 Mar 30;92(4):e02152-25. doi: 10.1128/aem.02152-25 (PMC13101533; doi:10.1128/aem.02152-25)
Supplement: Supplemental material — Fig. S1 to S7; Tables S1 and S2. [file aem.02152-25-s0001.pdf]

Supplementary data

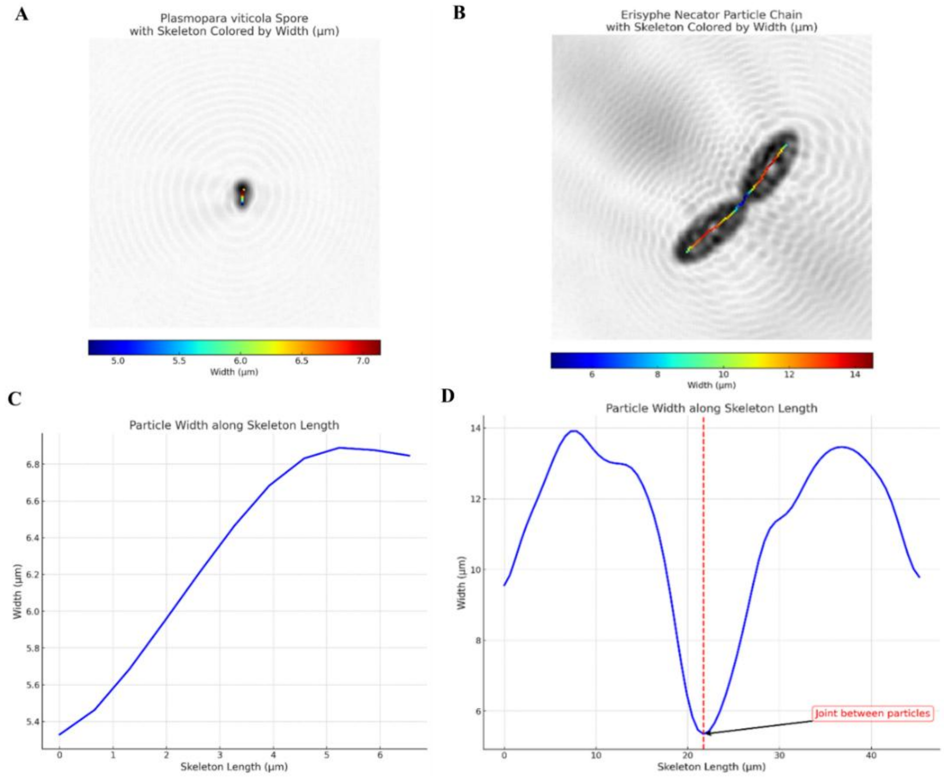

Fig. S1 Skeletonization strategy for precise classification of particles. (A) Holography image of *P. viticola* and (B) a chain of two *E. necator* fungal spores, with colored skeleton based on the particle width. (C) Particle width plotted against the skeleton length. (D) The minimum width marks the connection between the two fungal spores of *E. necator*.

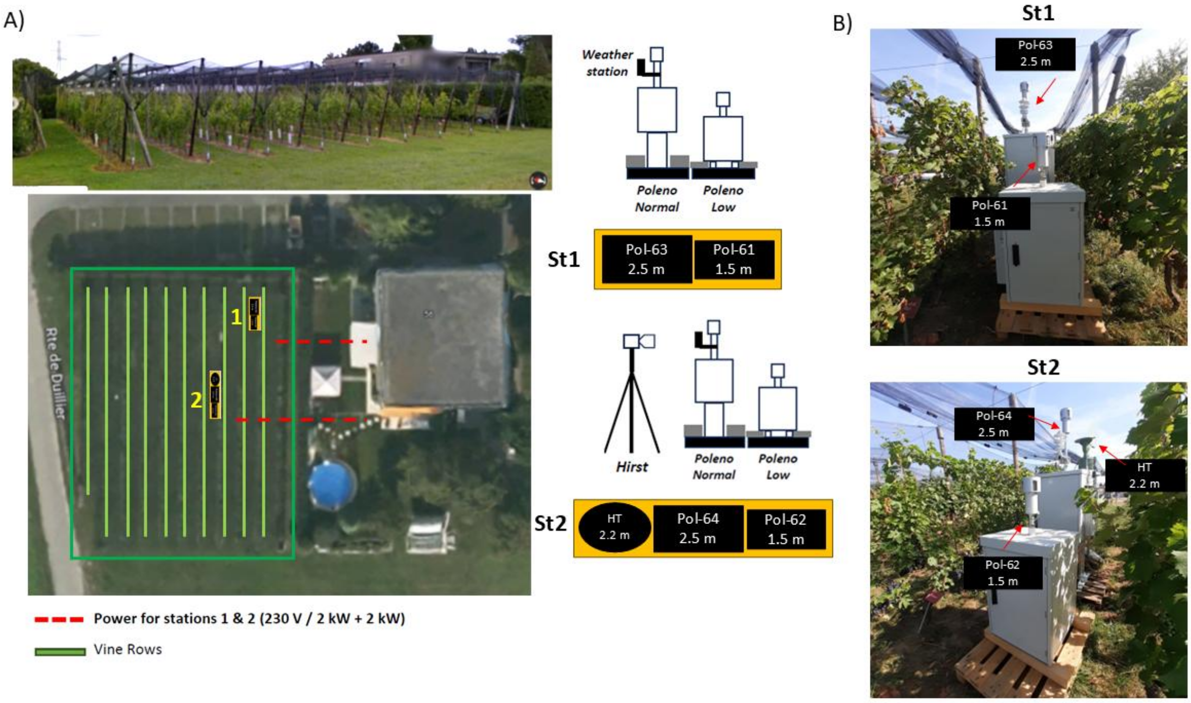

Fig. S2. Field experimental design of the SwisensPoleno pilot test in 2023. Four SwisensPoleno Jupiter instruments (Pol-61, Pol-62, Pol-63, Pol-64) were deployed in the collection vineyards of Changins College for Viticulture and Enology (Nyon, Switzerland). The devices were installed at two stations (St1 and St2), with two aerosol inlet heights tested at each station (1.5 m and 2.5 m). In addition, a Hirst-type spore trap (HT) was installed at St2. (A) Map and Street View imagery of Changins Nyon, Switzerland showing the experimental vineyard location [Map and Google Street View images], Google Maps (2023). Also, a schematic representation of the instruments used in the 2023 pilot test is presented. (B) SwisensPoleno device installed in the vineyard during the 2023 test.

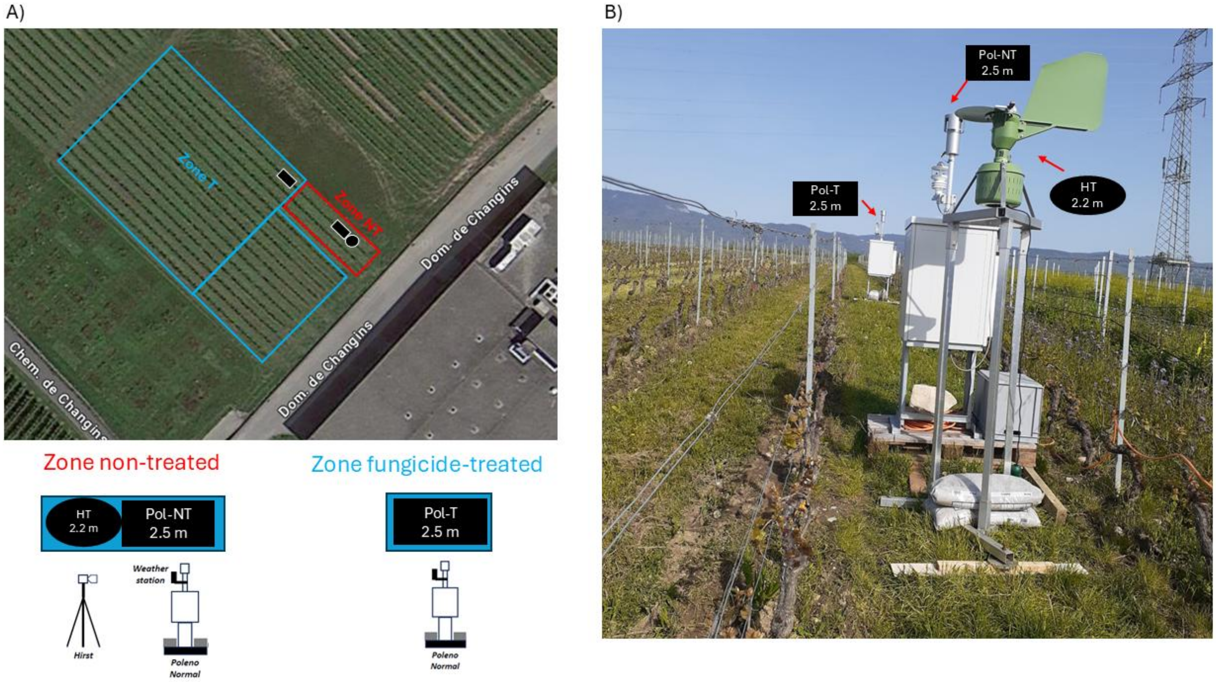

Fig. S3. Field trial setup of SwisensPoleno in the 2024 season. Two zones of an experimental vineyard at Changins (Nyon, Switzerland) were used to install two SwisensPoleno (Pol) instruments. The non-treated (NT) zone hosted Pol-NT together with a Hirst-type spore trap (HT), while the fungicide-treated (T) zone contained only Pol-T. (A) Satelit view of Nyon, Switzerland showing the vineyard located in Changins. Google Maps (2024), together with the schematic representation of the instruments used in the 2024 trial. (B) Newly installed SwisensPoleno sensors in the experimental vineyard for 2024 trial.

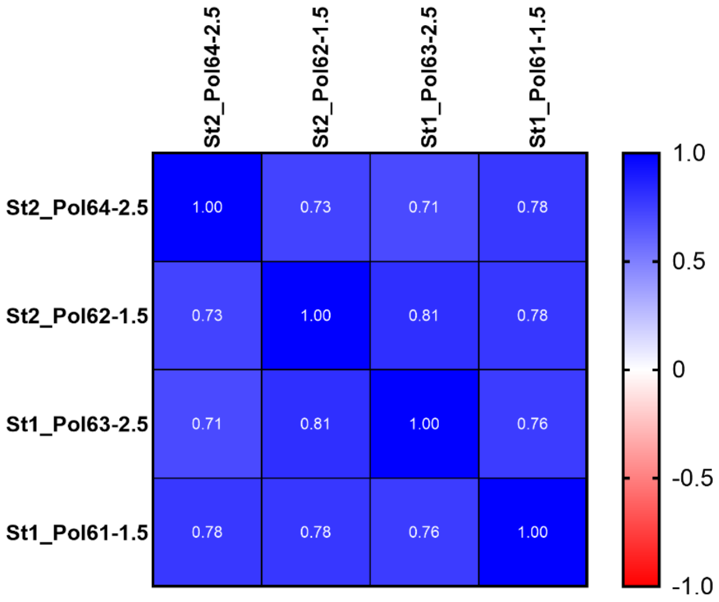

Fig. S4. Correlation between SwisensPoleno instrument measurements. Daily concentrations of *P. viticola* (sporangia/m^{-3}; log-transformed) were used to assess the internal consistency of the automated systems during the 2023 pilot season. Spearman correlation coefficients were calculated ($p < 0.05$).

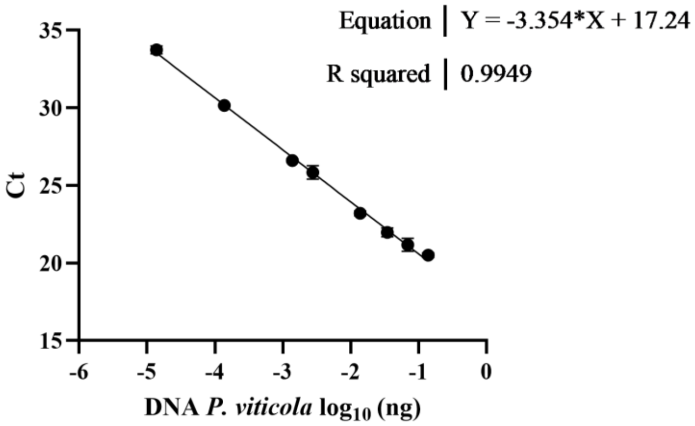

Fig. S5. Standard curve for *P. viticola* DNA qPCR quantification. Threshold cycles (Ct) were plotted against the log of genomic DNA standards of known concentrations. Each data point represents the mean of four replicates. Bars represent the standard error of the mean (SEM).

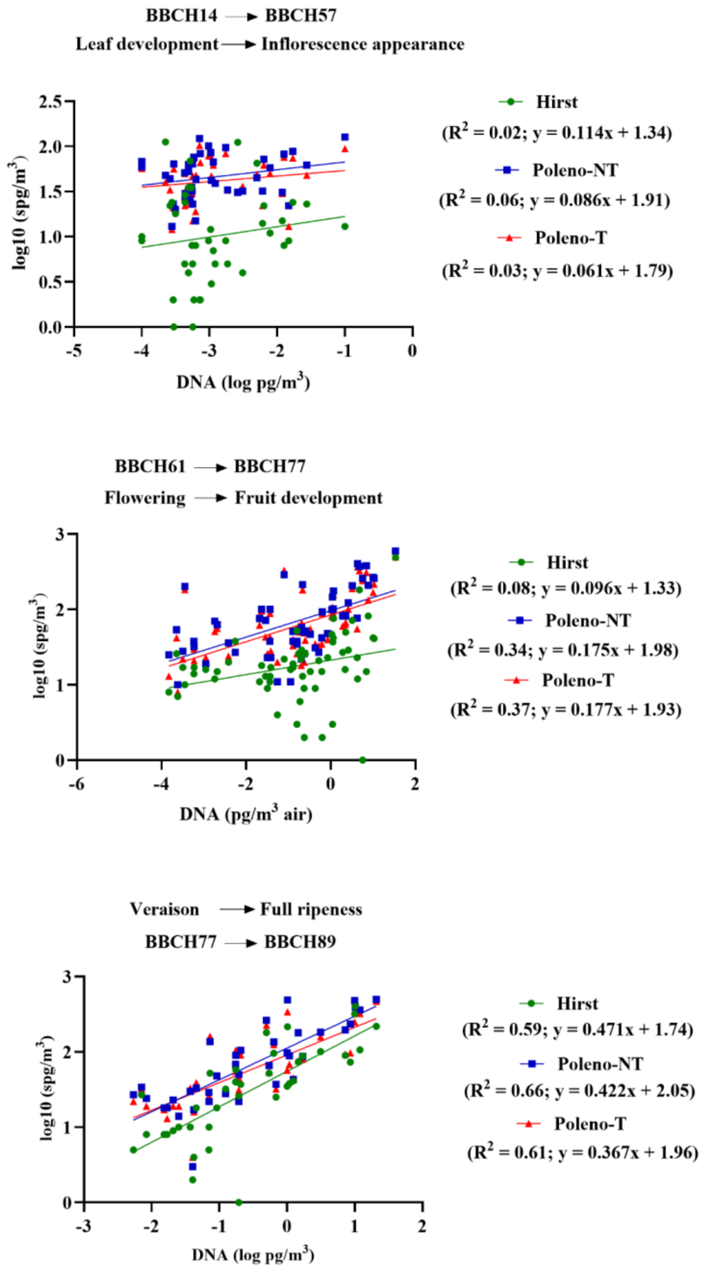

Fig. S6. Simple linear regression of SwisensPoleno measurements during the 2024 grapevine growing season. DNA quantification, and manual microscopic counting across vine phenological stages. Linear regressions ($p < 0.05$) were performed using log_{10}-transformed data from daily quantification of airborne *P. viticola* sporangia obtained by automatic classification with SwisensPoleno (Pol-NT and Pol-T), manual microscopic counts (Hirst), and molecular analyses (DNA). Datasets were divided according to the phenological stages described in Fig. 6.

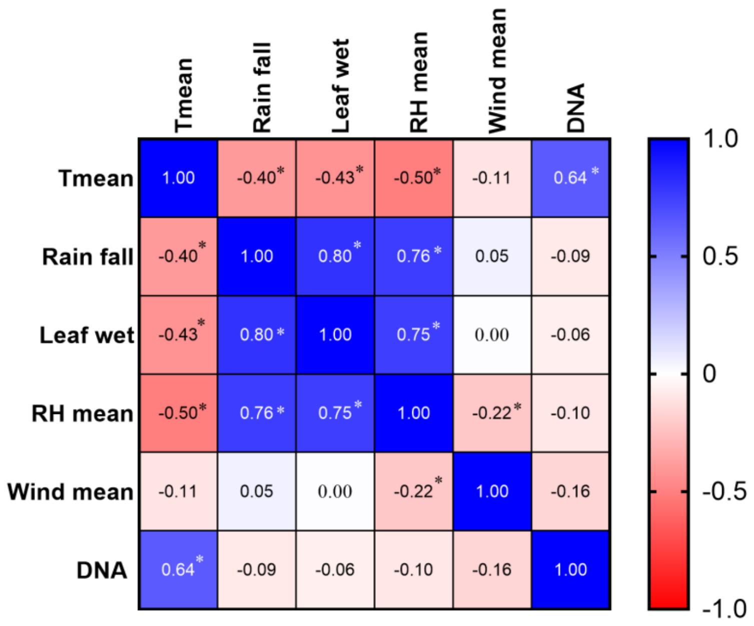

Fig. S7. Correlation between weather conditions and airborne DNA concentrations of *P. viticola* sporangia. Daily weather parameters - including mean temperature (Tmean, °C), mean wind speed (km/h), leaf wetness (h), mean relative humidity (RH, %), and rainfall (mm) - were compared with daily DNA concentrations (pg/m³ of air). Spearman correlations were calculated ($p < 0.05$).

Table S1. Sequence of Giop primes and probe used in this study.

Primer ID	Sequence 5'-3'(*)	Amplicon size/ 5'-3' modifications
PV_Giop_Fw	GGTTGCAGCTAATGGATTCCTA	208 bp
PV_Giop_Rv	TCCTGCAATTCGCATTACGT	
PV_Giop_ P-FAM	TCGCAGTTCGCAGCGTTCTTCA	5'-FAM & 3'-BHQ-1

(*) primer sequence obtained from Valsesia et al. 2005 and Si Ammour et al. 2020

Table S2. List of fungi used in the specificity test of qPCR assay for *P. viticola*

Fungi	Isolate code[a]	qPCR result
Fusarium graminearum	336	-
Aspergillus proliferans	1699	-
Penicillium chrysogenum	1575	-
Alternaria solani	113	-
Alternaria alternata	1554	-
Cladosporium sp.	2841	-
Drechslera ters	265	-
Epicoccum nigrum	2730	-
Botrytis cinerea	1612	-
Plasmopara viticola	FP 2024	+

[a]Codes refers to the culture collection of Mycology Research Group of Agroscope – Changins. FD: field population at the described year; - No amplification was detected via qPCR; + amplification detected via qPCR. The

underlined species represent other species in addition to those tested as off-target in the original primer design (Si Ammour et al. 2020)
